# A material change for ultra-high precision force sensing

**DOI:** 10.1038/s41377-024-01626-8

**Published:** 2024-09-26

**Authors:** Christopher Perrella, Kishan Dholakia

**Affiliations:** 1 Centre of Light for Life and School of Biological Sciences, University of Adelaide, Adelaide, South Australia 5005 Australia; 2 ARC Centre of Excellence in Optical Microcombs for Breakthrough Science (COMBS), University of Adelaide, Adelaide, South Australia 5005 Australia; 3 SUPA, School of Physics and Astronomy, University of St Andrews, St Andrews, KY16 9SS Scotland

**Keywords:** Nanoparticles, Optical manipulation and tweezers, Optical sensors, Biophotonics

## Abstract

An original form of photonic force microscope has been developed. Operating with a trapped lanthanide-doped crystal of nanometric dimensions, a minimum detected force of the order of 110 aN and a force sensitivity down to 1.8 fN/$\sqrt{{\rm{Hz}}}$ have been realised. This opens up new prospects for force sensing in the physical sciences.

## Abstract (web summary)

A photonic force microscope has been developed with a trapped lanthanide-doped crystal detecting forces as small as 110 aN. This opens new prospects for force sensing in the physical sciences.

## Graphical abstract

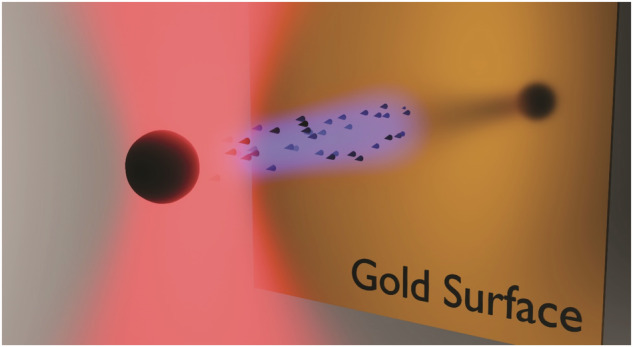

Measuring minute forces is at the heart of many studies across the physical and biological sciences. Stringent tests of fundamental physics are enabled by measuring minuscule forces. Within biology, forces play a fascinating role, and determining them is key to the burgeoning field of mechano-biology, with a suite of methods from the physical sciences advancing this field. Optical tweezers, generated by forces resulting from momentum transfer from a tightly focused light beam to a mesoscopic particle, is a prime example. This powerful method, recognised by the Nobel Prize in Physics in 2018, has allowed studies of single molecule biophysics^1^ such as DNA’s elastic parameters, and microrheometry within living cells^2^. Optical tweezers act as a Hookean spring at the micron scale, and can be converted into exquisite force transducers through precise measurement of a trapped particle’s position and appropriate calibration. Typically, optical tweezers trap an inert dielectric silica particle of approximately a micron in diameter as the probe. However, due to their ‘relatively’ large size, they inherently have a significant hydrodynamic drag and thus do not possess the spatiotemporal resolution to reveal fast dynamics such as molecular motor stepping dynamics^3^. Trapping sub-micron silica, or dielectric, particles is typically prohibited by damagingly high optical powers: the gradient force for successful confinement depends on polarizability which, in turn, scales with particle volume, thus rapidly diminishing when going from dielectric particles of micron size to nano-metre size^4^. However, exciting new opportunities arise by trapping sub-micron particles by invoking a material change in the probe particle itself which is at the heart of an exciting advance for force measurement, reported by Shan and colleagues in *Nature Photonics*^5^.

Optical tweezers is part of a broad and varied toolkit to probe forces in the biological world. Force transducers need to operate in physiological solutions at high spatial resolutions, but what is the scale of such forces and what approaches can we use to measure them? Contractile cellular forces drive bending, stretching, alignment, and repositioning of cells required for tissue development and homoeostasis, with forces on the scale of a nano-Newton. Traction forces during single cell migration are at the pico-Newton level. Cantilever-based force microscopy can resolve nano- to pico-Newton forces, but have limited three-dimensional (3D) spatial mapping capability. A recent demonstration by E. Dalaka et al. in *Light: Science and Applications* utilised deformable micro-lasers, in the form of dye-doped microdroplets, to enable spatial mapping of forces in the nano- to pico-Newton range deep (up to 300 microns) within Drosophila larvae^6^. Below the pico-Newton scale, we are in the realm of single molecule biophysics to explore studies such as the tension of a single DNA molecule^1^, and forces associated with kinesin^3^. Optical tweezers may be applied at this scale and can produce exquisite forces measurements into the 100fN range^3^, and have already elucidated many subtle and important issues in single molecule physics – quantifying some of the smallest forces measurable in the biological and chemical domains^7^.

Yet, how can we measure even smaller forces with optical tweezers? The breakthrough as shown by X. Shan et al.^5^ is by leveraging photonics with material science and advanced particle tracking. They describe a force measurement in aqueous solution with a minimum value of approximately 110aN. This extends optical tweezers beyond the femto-Newton^3,8–10^ range. What has held tweezers back from breaking this barrier until now? Issues have arisen when sensing below a femto-Newton force due to heating induced by high trapping power deteriorating biological samples. When reducing trap power to avoid this, one encounters low to undetectable scattering signals elevating localisation errors limiting the smallest measurable force that can be resolved.

To overcome these issues, the authors developed a super-resolved photonic force microscope (SRPFM), based on optical tweezers. At the heart of this advance, is the use of lanthanide-doped nanoparticles (Ln-NPs) of sizes ranging between 20 and 60 nm. Ln-NPs have only recently been applied to optical tweezers^11^, with a high trap strength, which is near two-orders of magnitude higher than traditional silica particles of comparable diameter. When compared to the commonly use gold nanoparticles, Ln-NPs achieve an impressive 30-fold higher optical trap strength. The high trap strength is due to a resonance effect that enhances the permittivity and polarizability of such a nanocrystal^11^. The high trap strength of Ln-NPs is leveraged within the SRPFM, allowing trapping with low optical powers of just over 10 mW. Importantly, this results in minimal sample heating of only 0.7 °C. This is a significant benefit when compared to utilising gold nanoparticles^12^ that, though offering high polarisability, experience heating due to laser absorption^13^. For silica, or dielectric, particles of sub-micron size, the optical powers required to maintain a stable trap are prohibitively high due to the optical gradient force scaling as described earlier. For both gold and dielectric nanoparticles, heating would thus significantly damage biological samples.

To utilise optical tweezers as a force transducer, one needs to precisely measure the particle’s position. To enable super resolution localisation of nanoparticle position, another benefit to the Ln-NPs becomes apparent: their bright emission. For biological imaging, Ln-NPs have enabled real-time particle tracking within living cells due to their bright emission^14^, thus avoiding high illumination intensities, minimising photobleaching and photodamage of biological samples^15^. This is critically important as the low optical trapping power also results in the reduced Ln-NP fluorescence making standard imaging approaches unsuitable for precise 3D particle localisation. Thus, Shan et al. drew upon concepts from 3D single-molecule localisation microscopy^16^. Imaging a Ln-NPs fluorescence upon a camera enabled determination of the particle’s transverse position via centroid fitting, however, the axial position remains ambiguous. This ambiguity is lifted utilising a cylindrical lens in the imaging path to introduce optical astigmatism into the point-spread-function of the imaged Ln-NP fluorescence, effectively encoding the axial position of the particle into its imaged ellipticity. To further improve the axial localisation, a deep neural network (DNN) was utilised to determine the axial position of the Ln-NP. Together, these methods achieve root-mean-squared localisation errors below 30 nm in the axial direction and 5 nm in the transverse directions, well below the diffraction limit of the Ln-NPs fluorescence and what is required to achieve sub femto-Newton force sensitivity.

For all optical tweezers, the smallest measurable force that can be resolved is fundamentally limited by fluctuating background forces exerted on the probe particle, often termed the thermal limit. For biological samples, this is typically the forces exerted by the thermal environment of the aqueous solution the probe particle is suspended within. Utilising the super resolution localisation, the authors track the Ln-NP with a localisation accuracy limited only by the Brownian motion of the particle, enabling demonstration of force measurements of 1.8 fN/$\sqrt{{\rm{Hz}}}$ at the thermal limit.

The authors demonstrate the performance of their advanced force microscope with two measurements; Firstly, the authors measure weak electrostatic forces acting upon individual Ln-NPs in the range of 108–781 aN produced via charged plates. In a second demonstration, the team measured the interaction force between a Ln-NP and a gold surface with a 80 fN force measured and attributed to a scattering-induced optical binding force^17^, illustrated in Fig. 1. Demonstration of such miniscule force measurements sets the scene for the use of Ln-NPs in spatially mapping of biological forces, a popular trend not limited just to optical tweezers^6^.

**Fig. 1:**
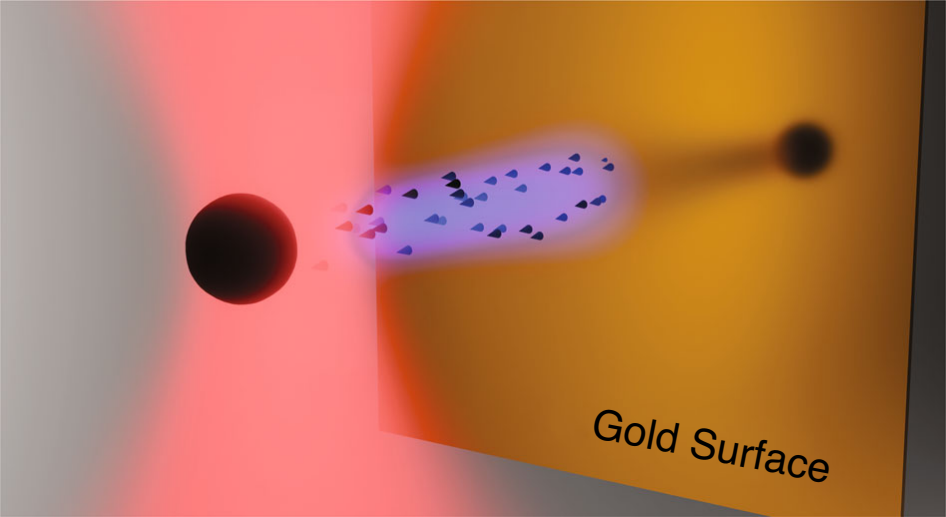
Illustration of a trapped Ln-NP (sphere) within an optical tweezers (red focused laser) forming the SRPFM. The Ln-NP is experiencing a repulsive scattering-induced optical binding force (blue) from the gold surface

The SRPFM has overcome major hurdles in force sensing, enabling measurements well below a femto-Newton resolution. This opens exciting possibilities in measuring molecular motors, conformational changes of proteins, cell patterning, micro-rheology, and adhesion forces. More broadly, optical tweezers are moving away from simple dielectric silica particles for the next generation of precision measurements. Recent work has used upconverting fluorescent nanoparticles for temperature sensing^18^, germanium nanospheres to make ultra-high resolution measurements of the motion of kinesin motors^3^, and rotating vaterite miro-spheres for measuring microrheometry within living cells^2^. The diversification of probe particles within optical tweezers, combined with innovative approaches to particle tracking, enables an array of new measurement possibilities. A material change is on the way for force measurement with optical tweezers.
